# Editorial: Anthony J. Thomas Jr.

**DOI:** 10.1590/S1677-5538.IBJU.2017.06.01

**Published:** 2017

**Affiliations:** Professor Titular Disciplina de Urologia da Faculdade de Medicina do ABC, Santo André, SP, Brasil Editor Chefe da International Braz J Urol

In November, 5th, 2017, on the day Dr. Anthony J. Thomas Jr. would turn 74, he passed away. He was an internationally recognized reputed urologist, that popularized microsurgery in the urological community.

Tony was a great friend of Brazilian Urology. He opened the doors of the Cleveland Clinic Foundation to Brazilian urologists. When he introduced me to his colleagues during meetings, he always stressed that I was his first fellow at Cleveland Clinic; he joined the Clinic in 1982, and worked there for more than 30 years. During that period, many other Brazilians attended fellowship under his guidance.

He always supported SBU and many times helped us bring several American experts to our Congresses without costs. He himself attended several Brazilian Congresses of Urology, and many national courses on Infertility.

But Tony's main quality was that he was a Family man. He involved his heart in all his actions. His patients firstly were persons that deserved his greater respect. He was a very thoughtful person, always with a nice and pleasant word for all. His concern with his fellows was touching. My car was very old, and he always would catch me when we had meetings together, since he was afraid that I would not make it due to mechanical problems. And I lived in the other side of the city! Probably he was the most honest, ethical and professional man that I have ever met.

In one occasion, we attended an Arabian prince with azoospermia due to epidydimal obstruction, that had been submitted to two surgical unsuccessful procedures. The year was 1983, without the possibility of testicular or epidydimal aspiration and ICSI. The boy's testicles looked like rocks, and Tony, very sadly, informed that it was impossible to reconstruct the tract satisfactorily. The very upset patient retorted that he would pay any amount of money for Tony to perform the surgery without assurance of success; Tony declined, because he could not offer any hope. When we left the office, the patient was crying and Tony's eyes were tearful. One week later, Tony received a golden watch with a card that simply stated: thank you for your honesty.

Talking to Tony was a pleasure; he always made you feel like the most important person in the world! I lived daily with him for almost one year and he had a huge influence in my career and in my way of being.

Humanity needed more people like Tony! What a pity!

Rest in Peace!

